# Associations between patient care ownership, burnout, and job satisfaction among medical residents: a nationwide cross-sectional study in Japan

**DOI:** 10.1038/s41598-026-40301-3

**Published:** 2026-02-14

**Authors:** Hirohisa Fujikawa, Hidetaka Tamune, Yuji Nishizaki, Kazuya Nagasaki, Hiroyuki Kobayashi, Masanori Nojima, Miwa Sekine, Taro Shimizu, Yu Yamamoto, Kiyoshi Shikino, Yasuharu Tokuda

**Affiliations:** 1 Department of General Medicine, Juntendo University Faculty of Medicine, 2-1-1 Hongo, Bunkyo-ku, Tokyo, 113-8421 Japan; 2 Center for General Medicine Education, School of Medicine, Keio University, Shinjuku-ku, Tokyo, Japan; 3 Department of Medical Education Studies, International Research Center for Medical Education, Graduate School of Medicine, The University of Tokyo, Bunkyo-ku, Tokyo, Japan; 4 Department of Psychiatry and Behavioral Science, Juntendo University Graduate School of Medicine, Bunkyo-ku, Tokyo, Japan; 5 Division of Medical Education, Juntendo University School of Medicine, Bunkyo-ku, Tokyo, Japan; 6 Department of Internal Medicine, Mito Kyodo General Hospital, University of Tsukuba, Mito, Ibaraki Japan; 7 Center for Translational Research, The Institute of Medical Science Hospital, The University of Tokyo, Minato-ku, Tokyo, Japan; 8 Department of Diagnostic and Generalist Medicine, Dokkyo Medical University Hospital, Mibu-machi, Shimotsuga-gun, Tochigi Japan; 9 Division of General Medicine, Center for Community Medicine, Jichi Medical University, Shimotsuke, Tochigi Japan; 10 Department of Community-Oriented Medical Education, Graduate School of Medicine, Chiba University, Chiba, Chiba Japan; 11 Muribushi Okinawa for Teaching Hospitals, Urasoe, Okinawa Japan; 12 Tokyo Foundation for Policy Research, Minato-ku, Tokyo, Japan

**Keywords:** Patient care ownership, Burnout, Job satisfaction, Well-being, Resident, Environmental social sciences, Health occupations

## Abstract

Although considerable bodies of literature have elucidated various determinants of burnout and job satisfaction among medical residents, one aspect that remains under-examined is the impact of patient care ownership (PCO). Therefore, we aimed to examine the associations between PCO, burnout, and job satisfaction among residents. This was a nationwide cross-sectional study, using online anonymous surveys from January 17 to March 31, 2024. The participants were residents who participated in the General Medicine In-Training Examination, a nationwide examination in Japan. The primary outcome was burnout, assessed using the Single-item Measure of Burnout Scale. The secondary outcome was job satisfaction, measured by the item included in the Mini-Z 2.0. The explanatory variable was PCO, as assessed by the Japanese version of the PCO Scale. 1816 participants were in the analysis. Multivariable logistic regression analyses revealed that, after adjustment for possible confounders, PCO showed a negative association with burnout, and that there was a positive association between PCO and job satisfaction. The findings suggest the importance of emphasizing PCO in the context of exploring burnout and job satisfaction among medical residents. This knowledge will inform educational interventions to help residents work with less burnout and greater job satisfaction, ultimately leading to better patient outcomes.

**Supplementary Information:**

The online version contains supplementary material available at 10.1038/s41598-026-40301-3.

## Introduction

Burnout of medical residents is prevalent and is a growing concern across the world^1^. Burnout is defined as the exhaustion, cynicism, and ineffectiveness that result from repeated exposure to emotional and interpersonal stressors in the workplace^2^. U.S. studies have shown that the burnout rate of physicians was high compared to that of the general population^3,4^. A literature review showed that among physicians, medical residents are particularly vulnerable to burnout, with a prevalence rate ranging from 27% to 75%, depending on specialty^5^. A Japanese study also found that approximately one-fifth to one-third of residents might experience burnout^6^. Residents are typically exposed to a substantial amount of stress, including demanding workload, financial problems, involvement in unfamiliar interprofessional team environments, and psychological and physical pressure from their supervisors and patients^7–10^. Resident burnout has undesirable effects on both residents themselves (e.g., increased risk of depression, suicidality, and medical illnesses)^11–13^and patients (e.g., greater risk of medical error and poorer quality of patient care)^14,15^. Thus, burnout of medical residents is a considerable issue that must be addressed internationally.

Job satisfaction has been extensively studied in fields other than medical education (economics, psychology, and sociology), and the results show that it can be associated with higher work productivity, less employee absenteeism, and lower employee turnover^16,17^. To date, few studies have elucidated the degree of job satisfaction among medical residents. However, these studies have nevertheless suggested the importance of job satisfaction, namely that higher job satisfaction among medical residents can lead to better patient care quality and more satisfied patients^18,19^. Accordingly, strategies to increase resident job satisfaction need to be investigated.

While substantial bodies of literature have explored various determinants of burnout and job satisfaction among medical residents, one aspect that remains understudied is the influence of patient care ownership (PCO). PCO is an essential component of medical professionalism and is defined as the cognitive-affective state that develops as a result of the individual’s knowledge of their patients, their management of these patients, and their emotional investment in a relationship with them^20–22^. PCO is essential for quality patient care^20,22^. Developing medical trainees’ PCO may lead to an improvement in their clinical skills and in patient outcomes^21^. Conversely, to our knowledge, no study has examined the association between PCO, burnout, and job satisfaction. Understanding this association is a priority, as it may provide insights into potential interventions and support systems to promote the well-being of medical residents.

We hypothesized that PCO would be negatively associated with burnout, and that PCO would be positively associated with job satisfaction. A scale of PCO, the PCO Scale (PCOS), has been validated^23,24^. In the U.S. validation study, correlational analysis without control for confounders indicated a significantly negative association between PCO and burnout, depressive condition, and frustration, and a significantly positive association between PCO and feeling energetic, happy, and fulfilled^23,24^. However, the U.S. study was limited by the fact that the association was tested using correlations only, and potential confounding variables were not adjusted. Nishigori et al. explored physician experiences of *yarigai* (a Japanese concept that encompasses the sense of fulfillment, intrinsic motivation, and satisfaction that comes from doing meaningful work)^25,26^in patient care^27^. The authors interviewed physicians who were recognized by their colleagues for their commitment to person-centered care and demonstrated *yarigai* in patient care. The qualitative analysis indicated that PCO led to positive emotions (e.g., a source of gratification and satisfaction, and a sense of belonging, respect, reward, and fulfillment)^27^. Thus, it is expected that there is a negative association between PCO and burnout, and a positive association between PCO and job satisfaction. However, excessive sense of responsibility can possibly lead to self-recrimination, distress, and burnout^28^. Therefore, studies employing a rigorous quantitative design to investigate these associations are necessary.

In Japan, medical care has long been sustained by the dedicated work of young physicians^29,30^, and depression and burnout among medical residents have been alarming problems^6,31^. Ogawa et al. showed that residents in Japan worked approximately 80 h per week, and that approximately 20% of residents exhibited newly developed depressive symptoms by the third month of residency^32^. As one response, the regulation of working hours was instituted in April 2024. Nevertheless, reducing medical resident burnout and increasing job satisfaction appear to be challenging and may require a multifaceted approach. These findings indicate the need for research that will lead to a deeper understanding of burnout and job satisfaction among medical residents, particularly in Japan.

Here, we examined the association between PCO and burnout among medical residents, with job satisfaction as a secondary outcome.

## Methods

### Postgraduate medical education system in Japan

To aid understanding of the context of this study, we here briefly summarize the postgraduate medical education system in Japan. After graduation from medical school and obtaining a medical license, medical trainees enter a two-year early residency program^33^. During the program, they rotate through several clinical departments^33^. After completing the program, they subsequently enter a three- to-five-year specialty training program^33^.

The General Medicine In-Training Examination (GM-ITE) serves as an objective measure of clinical knowledge of medical trainees in the early residency programs. The GM-ITE was first introduced in 2011 by the Japan Institute for Advancement of Medical Education Program (JAMEP), a nonprofit organization. It is modeled after the methodology of the U.S. Residency Internal Medicine In-Training Examination, ensuring its alignment with internationally recognized standards^34–36^. The GM-ITE is now a nationwide examination in which more than 50% of all medical residents in Japan participate^37,38^.

## Design, setting, and participants

This nationwide cross-sectional study was performed from January 17 to March 31, 2024. The study is a sub-study of a larger study of PCO. The other sub-studies include studies that examine the associations between PCO and other factors (e.g., amount of clinical knowledge)^37^.

We distributed an online, self-administered questionnaire to postgraduate year 1 (PGY-1) and PGY-2 medical residents in Japan who participated in the GM-ITE. Immediately following the GM-ITE, participants were asked to complete our questionnaire. Prior to participating in the study, all participants read the research document, which informed them that data would be anonymized, and that participation was voluntary. Only participants who agreed to participate in the study were included.

## Measures

### Primary outcome: burnout

We used the Japanese version of the Single-item Measure of Burnout Scale (SMB-J) to assess burnout^39^. While the Maslach Burnout Inventory (MBI) is the gold standard for assessing burnout^40^, its use is constrained by two major limitations. First, the inventory contains a relatively high number of items^39^, which can impose a burden on responders. Second, the license fee associated with its use is considerable^39^, which can be a significant financial obstacle for researchers. Given these challenges, the SMB has recently been given considerable attention as another measure of evaluating burnout^41^. The item is included in the Mini-Z 2.0, which is used to assess the well-being of physicians^42,43^. No license fee will be charged for use of SMB. The SMB consists of only one item; accordingly, the SMB and its translated versions are easy to administer and are widely used around the world. Nagasaki et al. examined the psychometric properties of the SMB-J and showed that it had the same level of diagnostic performance as the original SMB (sensitivity: 54%; specificity: 88%)^39^. They also found that SMB-J were strongly correlated with emotional exhaustion and cynicism subscales of the MBI-General Survey.

The single item is rated on a 5-point Likert scale, ranging from 1 = “I feel completely burned out. I am at the point where I may need to seek help.” to 5 = “I enjoy my work. I have no symptoms of burnout,” with lower scores indicating higher risk of burnout. A score of 3 or lower is defined as burnout^39,42,44^.

### Secondary outcome: job satisfaction

Job satisfaction was determined from the question “Overall, I am satisfied with my current job:”, which was rated on the following 5-point Likert scale: 1 = strongly disagree, 2 = disagree, 3 = neither agree nor disagree, 4 = agree, and 5 = agree strongly. The question is also included in the Mini-Z 2.0 and is used internationally^42,43^. For analysis, with reference to previous studies^45,46^, we decided to create a dichotomous variable for job satisfaction (not satisfied with job for “strongly disagree,” “disagree,” or “neither agree nor disagree”; and satisfied with job for “agree” or “agree strongly”).

### Explanatory variable: PCO

To assess PCO, the participants were asked to complete the questionnaire of the Japanese version of the PCOS (J-PCOS)^23,24,47^. The J-PCOS has been well-validated^47^. It has 13 items, each answered on a 7-point Likert scale from 1 (strongly disagree) to 7 (strongly agree)^47^. The 13-item measure includes 4 domains: assertiveness (6 items), sense of ownership (2 items), diligence (2 items), and being the “go-to” person (3 items)^47^. In this study, J-PCOS score was calculated as the mean of all 13 items. Scores for each domain (mean scores for items in each domain) were also employed. Both the J-PCOS score and its domain scores ranged from 1 to 7, with higher scores indicating a greater PCO.

### Covariates

Our regression models were adjusted for potential confounders. They were selected with reference to previous studies^5,24,48,49^, and included sex (female vs. male), PGY (PGY-1 vs. PGY-2), number of assigned inpatients (0–4; 5–9; 10–14; or ≥15), weekly working hours (< 50 h; ≥ 50 and < 55 h; ≥ 55 and < 60 h; ≥ 60 and < 70 h; ≥ 70 and < 80 h; ≥ 80 and < 90 h; or ≥ 90 h), hospital type (community hospital; university branch hospital; or university hospital), and hospital size (< 400 beds; 400–499 beds; 500–699 beds; or ≥ 700 beds).

### Statistical analysis

We first analyzed descriptive statistics. While continuous data were reported as means and deviations, categorical data were reported as frequencies and percentages.

We then checked for a clustering effect by calculating the intraclass correlation coefficient (ICC) to assess whether multilevel analysis was needed^50^. The ICC was below 10% for the outcome variables in the study. Accordingly, the clustering effect was considered small^50^, and we decided to perform a conventional analysis method. To elucidate the associations between PCO and burnout, and between PCO and job satisfaction, we used multivariable logistic regression analyses. As a sensitivity analysis, we also examined these associations using an ordered logistic regression analysis. For these analyses, we adjusted for possible confounders (sex, PGY, assigned inpatients, weekly working hours, hospital type, and hospital size). We chose complete case analysis. A two-tailed *p* < 0.05 was considered statistically significant.

The sample size for this study was not determined a priori. However, given the nature of the data collection process and the anticipated effect sizes, we believe that the sample size is sufficient to provide reliable and valid results. The large sample size of this study ensures adequate statistical power to detect meaningful differences or associations within the data. A previous study on a similar topic used comparable sample sizes^24^, further supporting the adequacy of our sample. For all statistical analyses, we used SPSS Statistics version 29.0 (IBM Corp).

### Ethical considerations

All methods were performed in accordance with the relevant guidelines and regulations. All study participants provided informed consent before participating. Ethical approval was obtained from the Ethics Review Board of the JAMEP (23 − 21).

## Results

9106 medical residents took the GM-ITE, of whom 2050 agreed to participate in our study. After excluding those with missing data, 1816 participants were included in the analyses.

Table 1 shows the characteristics of the 1816 participants. The majority were male (68.6%), in PGY-2 (50.9%), in charge of 5–9 inpatients (52.2%), and worked in a community hospital (81.1%). The participants’ responses to the J-PCOS are shown in the Supplementary Table 1.

**Table 1:** Characteristics of the study participants (*N* = 1816).

	Value
**Sex,** **n (%)** Female Male	571 (31.4) 1245 (68.6)
**PGY, n (%)** PGY-1 PGY-2	892 (49.1) 924 (50.9)
**Number of assigned inpatients, n (%)** 0–4 5–9 10–14 ≥ 15	671 (36.9) 948 (52.2) 151 (8.3) 46 (2.5)
**Weekly working hours, n (%)** < 50 h ≥ 50 and < 55 h ≥ 55 and < 60 h ≥ 60 and < 70 h ≥ 70 and < 80 h ≥ 80 and < 90 h ≥ 90 h	389 (21.4) 358 (19.7) 283 (15.6) 363 (20.0) 169 (9.3) 164 (9.0) 90 (5.0)
**Hospital type, n (%)** Community hospital University branch hospital University hospital	1472 (81.1) 113 (6.2) 231 (12.7)
**Hospital size, n (%)** < 400 beds 400–499 beds 500–699 beds ≥ 700 beds	506 (27.9) 385 (21.2) 553 (30.5) 372 (20.5)
**J-PCOS, mean (SD)** Total score Assertiveness Sense of ownership Diligence Being the “go-to” person	4.83 (0.99) 4.85 (1.03) 5.05 (1.08) 4.89 (1.12) 4.62 (1.17)
**SMB-J, n (%)** 1, 2, or 3 (= burnout) 4 or 5 (= not being in burnout)	264 (14.5) 1552 (85.5)
**Job satisfaction, n (%)** 1, 2, or 3 (= not being satisfied with job) 4 or 5 (= being satisfied with job)	401 (22.1) 1415 (77.9)

J-PCOS = Japanese version of the Patient Care Ownership Scale; PGY = postgraduate years; SD = standard deviation; SMB-J = Japanese version of the Single-item Measure of Burnout Scale.

J-PCOS scores and dimension scores range from 1 to 7, with higher scores indicating higher PCO. SMB-J scores range from 1 to 5, with a lower score indicating higher risk of burnout. The scores of the item measuring job satisfaction range from 1 to 5, with greater scores indicating more satisfied with job.

Table 2 shows the results of the multivariable logistic regression analyses. After adjustment for possible confounders, total J-PCOS score showed a significantly negative association with burnout (adjusted odds ratio per 1-point increase, 0.63; 95% confidence interval, 0.55–0.73). All domains of the J-PCOS (i.e., assertiveness, sense of ownership, diligence, and being the “go-to” person) were also significantly negatively associated with burnout. Total J-PCOS scores were significantly positively associated with job satisfaction (adjusted odds ratio per 1-point increase, 1.85; 95% confidence interval, 1.62–2.11), after adjustment for possible confounders. All domains of the J-PCOS also had a significant association with job satisfaction.

**Table 2:** Associations of PCO, burnout, and job satisfaction among medical residents (*N* = 1816).

	Unadjusted odds ratio (95% CI)^b^	Adjusted^a^ odds ratio (95% CI)^b^
**Burnout**		
**J-PCOS** ^**c**^ Total score Assertiveness Sense of ownership Diligence Being the “go-to” person	0.66 (0.58–0.76)* 0.68 (0.60–0.77)* 0.81 (0.72–0.91)* 0.71 (0.63–0.79)* 0.73 (0.65–0.82)*	0.63 (0.55–0.73)* 0.65 (0.57–0.75)* 0.79 (0.70–0.90)* 0.69 (0.62–0.78)* 0.71 (0.63–0.80)*
**Job satisfaction**		
**J-PCOS** ^**c**^ Total score Assertiveness Sense of ownership Diligence Being the “go-to” person	1.88 (1.66–2.14)* 1.78 (1.58–2.01)* 1.51 (1.36–1.68)* 1.63 (1.47–1.81)* 1.56 (1.41–1.73)*	1.85 (1.62–2.11)* 1.75 (1.55–1.99)* 1.50 (1.34–1.67)* 1.59 (1.43–1.77)* 1.54 (1.39–1.71)*

CI = confidence interval; J-PCOS = Japanese version of the Patient Care Ownership Scale; PCO = patient care ownership.

* *p* < 0.001.

^a^ Adjusted for sex, postgraduate years, the number of assigned patients, weekly working hours, hospital type, and hospital size.

^b^ Per 1-point increase.

^c^ All scores range from 1 to 7.

For robustness checks, we also conducted the ordered logistic regression analysis to elucidate the associations between total J-PCOS score, burnout, and job satisfaction. The results of this sensitivity analysis were consistent with those of the main analysis (Supplementary Table 2).

## Discussion

This nationwide cross-sectional study of Japanese medical residents found that PCO assessed by J-PCOS was negatively associated with burnout, and that PCO was positively associated with job satisfaction. In addition, all domains of the J-PCOS showed a negative association with burnout and a positive association with job satisfaction. Our findings will aid a deeper understanding of the issues of burnout, job satisfaction, and PCO.

The mean PCO score for our cohort (4.83) was lower than that of the U.S. cohort (5.57)^23^. From an educational perspective, the mean value difference of approximately 1 point between the two countries appears to be significant. For example, we believe that the difference between 4 = “Neither agree nor disagree” and 5 = “Somewhat agree” for the item “I felt a strong sense of ownership of my patients’ care” is meaningful. The score differences are likely attributable to differences in the postgraduate medical education system. The U.S. study included internal medicine residents of PGY 1–3^23^. Conversely, as stated in the Methods section, the early residency program in Japan consists of PGY 1–2 residents. In the system, where residents rotate through various departments in a short period of time, there is often less patient continuity, and residents may face challenges in developing PCO. We will consider how to further clarify the mechanism of the score differences in our future studies. Additionally, future studies that examine the interpretability of the PCOS would be insightful.

The SMB-J burnout rate of 14.5% in this study was low in comparison to the prevalence of burnout in residents as presented in the Introduction section^5,6^. We propose several reasons for this. First, it is possible that medical residents with greater risk of burnout were less likely to participate in the study. This phenomenon may be further influenced by the reluctance of Japanese individuals to disclose their own mental health issues^51^. Second, the sensitivity of the SMB is relatively low. Nagasaki et al. showed that the sensitivity of the SMB-J for detecting burnout was approximately 50%^39^.

As noted in the Introduction, some studies have explored the link between PCO, burnout, and job satisfaction. However, these studies had several methodologic limitations. The U.S. study aimed to validate the PCOS and explored the relationship between PCO and burnout using correlational analysis only^23,24^. The Japanese study qualitatively explored the concept of *yarigai*, which identified a potential association between PCO and job satisfaction^27^. Thus, this study is the first to combine the three concepts of PCO, burnout, and job satisfaction in a robust quantitative study design.

The importance of this study is highlighted by the ongoing global prevalence of burnout among medical residents, which has an undesirable impact on residents. Our findings indicate the significance of emphasizing PCO in the context of exploring resident burnout and job satisfaction. Given the current prevalence of burnout, changes in medical professionalism are now taking physician well-being into account^52,53^, and countries across the world, including Japan, are regulating physician work hours^54^. Conversely, as Nishigori et al. suggested, it is “the time to approach medical professionalism from the perspective of physician satisfaction and consider the meaning of working as a doctor”^27^. If resident physicians take responsibility for patient care autonomously and find *yarigai* in patient care, work may become more than a means of earning money, but rather a source of fulfillment^27^, resulting in less burnout and greater job satisfaction. The question then becomes how can we foster PCO among resident physicians? A recent Canadian study revealed that it was crucial to promote a supportive learning environment with positive role modeling to foster medical trainees’ PCO^55^. Soeprono et al.’s qualitative study suggested possible educational interventions to develop PCO, including setting expectations, modeling, promoting autonomy, supervising countertransference, changing culture of residency programs, and longer clinical rotations^56^. It may also be helpful to listen to the *yarigai* stories of experienced physicians and discuss them with peers^27^. Our future research plan is to test whether these interventions aimed at developing PCO really lead to a reduction in burnout and improvement in job satisfaction.

To our knowledge, the present study is the first to report a negative relationship between PCO and burnout, and a positive association between PCO and job satisfaction. A key strength of this study was the use of data from a nationwide study conducted on GM-ITE takers, representing half of all medical residents in Japan. In addition, the assessment tool used, the J-PCOS, is a well-validated and widely utilized measure for assessing PCO^23,24,47^. Thus, our study has relatively high external validity. On the other hand, the study was conducted in a single Asian country and exclusively focused on early medical residents. Given mounting recognition of the importance of burnout and job satisfaction, future studies should include physicians of PGY 3 or above and those from beyond Asia, which would provide international medical educators with valuable insights.

### Limitations

Our study had some potential limitations. First, it was performed under a cross-sectional design. On one hand, a timeline of 3 months as study/analysis duration might fit in the cross-sectional domain, it might at the same time present challenges in the drawing of conclusions on burnout and satisfaction based on PCO. Further longitudinal studies are needed to confirm causality and direction. Second, we calculated the odds ratio in this study. The duration of the study was relatively long. The population being studied was considered representative of the total resident physician volume, as the GM-ITE is a nationwide examination taken by more than half of all medical residents in Japan. Thus, the decision was made to employ the odds ratio with consideration to the use of multivariable logistic regression analysis, but it is possible that this decision led to overestimation. Third, the study used simple measures to assess burnout and job satisfaction. Because the questionnaire was conducted immediately after the GM-ITE, we had to reduce the number of the survey items. Nevertheless, the items used to assess burnout and job satisfaction are both included in the Mini-Z 2.0 and are widely used^42,43^, with the SMB-J in particular having excellent specificity despite being a single item^39^. Future options include the use of other measures of burnout, particularly the MBI, which comprises the three domains of emotional exhaustion, depersonalization, and personal accomplishment^57^. In contrast, the SMB captures the emotional exhaustion domain only^58^. Given that the depersonalization and personal accomplishment domains appear to be in greater opposition to PCO than the emotional exhaustion domain, we expect a stronger inverse association of PCO and burnout when assessed by the MBI. Fourth, although we selected covariates with reference to literature, unknown confounders can affect the results. For example, supportive relationships with colleagues or a positive work environment can be associated with greater PCO, less burnout, and higher job satisfaction^59^. Future studies should consider these potential confounding factors. Fifth, there is a potential for selection bias. In our study, the proportion of medical residents affiliated with a university hospital was limited to less than 20%, although university-affiliated medical residents comprise about half of all residents in Japan. In addition, as noted above, the rate of burnout was lower in the study, which also raised concerns about potential selection bias. Accordingly, caution is required when generalizing the results. Sixth, the response rate was a concern. The relatively low response rate may be attributable to several causes. One potential explanation is that we conducted this study based on a survey at the time when medical residents took their in-training examinations. This circumstance may have led to a decline in residents’ motivation to engage with the study. An alternative hypothesis is that medical trainees with less PCO, greater risk of burnout, or less job satisfaction were less likely to complete our survey questionnaire. If so, this response trend might have caused underestimation of the association between PCO and burnout, and between PCO and job satisfaction. Seventh, the study was conducted just prior to the implementation of physician working hour regulations. In the future, PCO, burnout, and job satisfaction among medical residents may be affected by implementation of the new policy. Comparison of results before and after the new policy implementation may further deepen our knowledge of this area.

## Conclusions

This study of medical residents revealed that there was a negative association between PCO and burnout, and a positive association between PCO and job satisfaction. Our findings will aid a deeper understanding of burnout, job satisfaction, and PCO, and will be helpful for preventing burnout and promoting job satisfaction among resident physicians.

## Supplementary Information

Below is the link to the electronic supplementary material.

Supplementary Material 1

Supplementary Material 2

## Data Availability

Only upon reasonable request, the corresponding author can provide the data sets generated and analyzed in the present study.
