# Supplementary material for: Associations between patient care ownership, burnout, and job satisfaction among medical residents: a nationwide cross-sectional study in Japan
Source: Sci Rep. 2026 Feb 14;16:9119. doi: 10.1038/s41598-026-40301-3 (PMC12996442; doi:10.1038/s41598-026-40301-3)
Supplement: Supplementary file 1 — Supplementary Material 1 [file 41598_2026_40301_MOESM1_ESM.docx]

**Supplementary file 1**

**Supplementary Table 1: Participants’ responses to the Japanese version of the Patient Care Ownership Scale**

| **Item** | **Factor^a^** | **Responses^b^, n (%)** | | | | | | | **Mean** | **Standard deviation** |
| --- | --- | --- | --- | --- | --- | --- | --- | --- | --- | --- |
|  |  | **1** | **2** | **3** | **4** | **5** | **6** | **7** |  |  |
| Q1. I was vocal and assertive about my patients’ best treatment/care. | F1 | 8 (0.4) | 21 (1.2) | 54 (3.0) | 260 (14.3) | 686 (37.8) | 571 (31.4) | 216 (11.9) | 5.30 | 1.07 |
| Q2. I was the “go-to” person for knowledge about my patients. | F4 | 38 (2.1) | 112 (6.2) | 189 (10.4) | 568 (31.3) | 482 (26.5) | 293 (16.1) | 134 (7.4) | 4.52 | 1.36 |
| Q3. I was proactive in checking up on my patients, rather than being called with questions or concerns. | F4 | 18 (1.0) | 39 (2.1) | 146 (8.0) | 445 (24.5) | 656 (36.1) | 359 (19.8) | 153 (8.4) | 4.86 | 1.20 |
| Q4. I ensured good continuity of care even when I was absent from the service. | F1 | 15 (0.8) | 44 (2.4) | 99 (5.5) | 463 (25.5) | 641 (35.3) | 390 (21.5) | 164 (9.0) | 4.93 | 1.18 |
| Q5. I felt comfortable telling the attending what I felt was the right thing to do for my patients, rather than just letting them decide. | F1 | 18 (1.0) | 49 (2.7) | 154 (8.5) | 363 (20.0) | 643 (35.4) | 409 (22.5) | 180 (9.9) | 4.93 | 1.25 |
| Q6. I made sure that the nursing staff was updated with the day’s plan. | F4 | 52 (2.9) | 121 (6.7) | 234 (12.9) | 481 (26.5) | 490 (27.0) | 306 (16.9) | 132 (7.3) | 4.48 | 1.42 |
| Q7. I was given the opportunity to make decisions independently about my patients’ care. | F1 | 26 (1.4) | 56 (3.1) | 96 (5.3) | 340 (18.7) | 678 (37.3) | 429 (23.6) | 191 (10.5) | 5.00 | 1.25 |
| Q8. I personally made sure to go back and check that all orders were actually carried out. | F3 | 27 (1.5) | 84 (4.6) | 203 (11.2) | 382 (21.0) | 637 (35.1) | 344 (18.9) | 139 (7.7) | 4.71 | 1.31 |
| Q9. When carrying out my patient’s management plan, I took extra care to make sure that things did not fall through the cracks. | F3 | 13 (0.7) | 33 (1.8) | 88 (4.8) | 326 (18.0) | 745 (41.0) | 445 (24.5) | 166 (9.1) | 5.07 | 1.12 |
| Q10. I felt comfortable making decisions independently about my patients’ care. | F1 | 31 (1.7) | 66 (3.6) | 172 (9.5) | 443 (24.4) | 637 (35.1) | 327 (18.0) | 140 (7.7) | 4.72 | 1.28 |
| Q11. I challenged the team as needed if I felt it was in my patients’ best interest, no matter how much push back I got. | F1 | 71 (3.9) | 164 (9.0) | 282 (15.5) | 539 (29.7) | 400 (22.0) | 238 (13.1) | 122 (6.7) | 4.23 | 1.47 |
| Q12. I felt responsible for my patients’ care, even after my shift ended. | F2 | 26 (1.4) | 52 (2.9) | 100 (5.5) | 369 (20.3) | 694 (38.2) | 394 (21.7) | 181 (10.0) | 4.96 | 1.24 |
| Q13. I felt a strong sense of ownership of my patients’ care. | F2 | 11 (0.6) | 22 (1.2) | 64 (3.5) | 340 (18.7) | 726 (40.0) | 460 (25.3) | 193 (10.6) | 5.15 | 1.09 |

^a^ Factor 1 = Assertiveness; Factor 2 = Sense of ownership; Factor 3 = Diligence; Factor 4 = Being the “go-to” person

^b^ 1 = Strongly disagree; 2 = Disagree; 3 = Somewhat disagree; 4 = Neither agree nor disagree; 5 = Somewhat agree; 6 = Agree; 7 = Strongly Agree
