# Supplementary material for: Associations between patient care ownership, burnout, and job satisfaction among medical residents: a nationwide cross-sectional study in Japan
Source: Sci Rep. 2026 Feb 14;16:9119. doi: 10.1038/s41598-026-40301-3 (PMC12996442; doi:10.1038/s41598-026-40301-3)
Supplement: Supplementary file 2 — Supplementary Material 2 [file 41598_2026_40301_MOESM2_ESM.docx]

**Supplementary file 2**

**Supplementary Table 2: Results of the ordered logistic regression analysis that examined the associations between patient care ownership, burnout, and job satisfaction**

|  | Unadjusted odds ratio (95% CI)^b^ | Adjusted^a^ odds ratio (95% CI)^b^ |
| --- | --- | --- |
| Burnout^c^ |  |  |
| J-PCOS^e^  Total score | 1.76 (1.60–1.94)* | 1.82 (1.64–2.02)* |
| Job satisfaction^d^ |  |  |
| J-PCOS^e^  Total score | 2.11 (1.92–2.33)* | 2.13 (1.92–2.35)* |

* p < 0.001

^a^ Adjusted for sex, postgraduate years, the number of assigned patients, weekly working hours, hospital type, and hospital size.

^b^ Per 1-point increase

^c^ Burnout is rated on a 5-point Likert scale, ranging from 1 = “I feel completely burned out. I am at the point where I may need to seek help.” to 5 = “I enjoy my work. I have no symptoms of burnout.”

^d^ The scores of the item measuring job satisfaction range from 1 to 5, with greater scores indicating more satisfied with job.

^e^ The scores range from 1 to 7.
